# Molecular characterization of *Bacillus thuringiensis* using rep-PCR

**DOI:** 10.1186/2193-1801-2-641

**Published:** 2013-11-29

**Authors:** Rosane Bezerra da Silva, Fernando Hercos Valicente

**Affiliations:** Department of Plant Biotechnology, Federal University of Lavras, Lavras, Minas Gerais Brazil; Department of Biological control, Embrapa Maize and Sorghum research, Sete Lagoas, Minas Gerais Brazil

**Keywords:** REP-PCR, Genetic divergence, *Bacillus thuringiensis*, Repetitive sequences

## Abstract

The genetic divergence of 65 strains of *Bacillus thuringiensis* (Bt) was determined using Rep-PCR*.* Based on the repetitive sequences the BOX primer was the most informative with 26 fragments, followed by ERIC (19) and REP (10), generating a total of 55 fragments. The dendogram shows that ten groups were formed when 45% was the average distance of the population: group 1 with 41,5% of the isolates, 33,8% of the isolates were distributed in other groups and 24,6% did not formed distinct group. 53,2% of the isolates from Embrapa are in the group 1, and 29,8% of the isolates are distributed in other groups. Bt strains from USDA and Institute Pasteur showed more variability.

## Introduction

*Spodoptera frugiperda* (Smith) is responsible for significant losses in maize yield in Brazil. Its control is mainly achieved using chemical insecticides. However, biological control using *Bacillus thuringiensis* Berliner (Bt), and the production of genetically modified plants with insect resistance genes are promising alternatives to control these insect (Valicente and Barreto, 2003).

*B. thuringiensis* (Bt) is a gram-positive bacterium that produces protein crystalline inclusions called Cry proteins during the stationary phase encoded by different *cry* genes (Yamamoto & Dean, 2000). *B. thuringiensis* can be found in different substrates such as soil, water, plant surfaces, dead insects, grain dust, spider webs and stored grain (Glare & O’Callaghan 2000; Valicente & Barreto 2003; and Miralles & Peres 2004). This bacterium also shows a high genetic variability and is widely distributed in nature (Vilas-Boas et al. 2002). The genetic variability between different isolates has been studied using the polymerase chain reaction (PCR) with positive results (Shangkuan et al. 2001; Lima et al. 2002).

However, Repetitive Element Polymorphism REP-PCR fingerprinting has become a frequent method to discriminate bacteria species analyzing the distribution of repetitive DNA sequences in several prokaryotic genome (Versalovic et al. 1991). It is reliable, reproducible and simple, and of rapid implementation, in addition to high efficiency with the discrimination of microorganisms, even among populations of the same species (Versalovic et al. 1994; Rademaker & De Bruijin 1997; Louws et al. 1999).

REP-PCR is based on the observation that outwardly facing oligonucleotide primers, complementary to interspersed repeated sequences, enable the amplification of differently sized DNA fragments, consisting of sequences lying between these elements (Versalovic et al. 1994). Multiple amplicons of different sizes can be resolved by electrophoresis, establishing DNA fingerprint specific patterns for bacteria strains (Rademaker & De Bruijin 1997). Several of these interspersed repetitive elements are conserved in diverse genera of bacteria and, therefore, enable single primer sets to be used for DNA fingerprinting in many different microorganisms (Versalovic et al. 1994; Rademaker & De Bruijin 1997). Moreover, it is not necessary prior knowledge of the genomic sequence of a species, with an initial selection of primers, to have this approach generates results in a short period of time (Shangkuan et al. 2001; Lima et al. 2002).

The Palindromic Units (PU) Repetitive Extragenic Palindromes (REP) constitutes the characterized family of bacterial repetitive sequences. PU are present in about 500–1000 copies in the chromosome of *Escherichia coli* and of *Salmonella typhimurium.* PU sequences consist of a 35–40 bp inverted repeat and are found in clusters in which successive copies (up to six) are arranged in alternate orientation (Higgins et al. 1982; Gilson et al. 1984; Martin et al. 1992).

A second family of repetitive elements, called IRU (Intergenic Repeat Units) or ERIC (Enterobacterial Repetitive Intergenic Consensus), has been described (Versalovic et al. 1991). IRU are 124–127 bp long and are present in about 30–50 copies in *E. coli* and 150 copies in *S. typhimurium*. Although IRU resembles PU in several features, the nucleotide sequence is entirely different and PU IRU, appear to occur singly. Both PU and IRU families are similarly located in non-coding, probably transcribed, regions of the chromosome. The consensus BOX element is constituted, from 5′ to 3′, of three subunits, boxA (59 bp), boxB (45 bp), and boxC (50 bp), and it is 154 bp long present in about 25 fragments of the *S. pneumoniae* chromosome (Stern et al. 1984; Sharples & Lloyd 1990; Versalovic et al. 1991; Martin et al. 1992; Shuhaimi et al. 2001).

Versalovic et al. (1991) outlined pairs of primer sequences corresponding to ERIC, REP and BOX palidromic sequences. However, little is known about the efficiency of these primers to give information about *B. thuringiensis* isolates. This study aimed to estimate the genetic diversity of 65 strains of *B. thuringiensis* based on ERIC, REP and BOX sequences, and identifies possible groupings related subspecies.

## Materials and methods

Of a total of 65 *B. thuringiensis* strains (Table 1), 26 were identified as: nine kindly provided by the USDA (United States Department of Agriculture), 9 kindly provided by the Institute Pasteur, 8 from Embrapa Maize and Sorghum Bt Bank, and 39 strains with no subspecies information also from Embrapa’s collection. All strains have been previously tested against fall armyworm, *S. frugiperda* (Lepidoptera: Noctuidae J.E. Smith) (Valicente & Fonseca 2004; Valicente & Barreto 2003). These strains are stored in glycerol at – 20°C.

**Table 1:** ***Bacillus thuringiensis*** **serovars used in this study**

N°	Strain identification	Mortality (%)	Origin	N°	Strain identification	Mortality (%)	Origin
**1**	HD-4 *Bt alesti*	6.8	USA	**34**	1109 N	100	Goiás
**2**	348B – *Bt alesti*	100	Paraná	**35**	1145B	100	Goiás
**3**	T-07 *Bt aizawai*	80.8	France	**36**	1145C	100	Goiás
**4**	HD-11 *Bt aizawai*	7.8	USA	**37**	1148 F	100	Goiás
**5**	462A– *Bt galleriae*	100	Paraná	**38**	1132E	100	Goiás
**6**	474 – *Bt galleriae*	100	Paraná	**39**	1135B	100	Goiás
**7**	348 L – *Bt galleriae*	100	Paraná	**40**	1136B	100	Goiás
**8**	HD-29 *Bt galleriare*	12.8	USA	**41**	1139 K	100	Goiás
**9**	344 – *Bt tolworthi*	100	Paraná	**42**	BTLM	100	Goiás
**10**	T-09 *Bt tolworthi*	100	France	**43**	1644	100	Paraná
**11**	426 – *Bt tolworthi*	100	Ceará	**44**	1641	100	Paraná
**12**	461A – *Bt tolworthi*	100	Paraná	**45**	701A	100	São Paulo
**13**	460 – *Bt darmstadiensis*	100	Paraná	**46**	1658	100	São Paulo
**14**	T-10 *Bt darmstadiensis*	77.9	France	**47**	1646	100	São Paulo
**15**	T-06 *Bt entomocidus*	9.8	France	**48**	701B	100	São Paulo
**16**	T-24 *Bt neoleonensis*	17.9	France	**49**	1648	100	São Paulo
**17**	T-27 *Bt mexicanensis*	17	France	**50**	1438	100	Sergipe
**18**	T-23 *Bt japonensis*	33.5	France	**51**	1438H	100	Sergipe
**19**	T-16 *Bt indiana*	12.2	France	**52**	1089E	95.8	Minas Gerais
**20**	HD-73 *Bt kurstaki*	2.7	USA	**53**	1091H	95.8	Minas Gerais
**21**	HD-1 *Bt kurstaki*	0	USA	**54**	1604D	95.2	Amazônia
**22**	HD-12 *Bt morrisoni*	28	USA	**55**	1605	95.2	Amazônia
**23**	HD-7 *Bt dendrolimus*	5.4	USA	**56**	1657	100	Amazônia
**24**	HD-3 *Bt finitimus*	5.2	USA	**57**	1656	100	Alagoas
**25**	HD-2 *Bt thuringiensis*	37.8	USA	**58**	1603B	100	Santa Catarina
**26**	T-14 *Bt israelensis*	0	França	**59**	1626C	97.6	Maranhão
**27**	1119C	100	Goiás	**60**	702	97.6	Mato Grosso
**28**	1124E	100	Goiás	**61**	1354	100	Minas Gerais
**29**	1131A	100	Goiás	**62**	1355SLO	100	Minas Gerais
**30**	1131C	100	Goiás	**63**	1357E	100	Paraná
**31**	1132A	100	Goiás	**64**	566	100	Paraná
**32**	1132C	100	Goiás	**65**	1355LM	100	Minas Gerais
**33**	1138G	100	Goiás				

### DNA extraction and PCR conditions

Genomic DNA from Bt strains was isolated and purified according to Shuhaimi et al. (2001).

ERIC and BOX primers:

The amplification reactions were conducted using 30 ng DNA, 3 mM MgCl2, 20 mM Tris pH 8.0, 50 mM KCl, 250 μM of dNTP, 10 μM of each primer and two units of Taq DNA polymerase, in a final volume of 20 μl.

The sequences of primers designed by Versalovic et al. (1994) were:

- ERIC 1 5′- ATGTAAGCTCCTGGGGATTCAC-3′
- ERIC 2 5′- AAGTAAGTGACTGGGGTGAGCG-3′
- BOX 5′-CTACGGCAAGGCGACGCTGACG-3

The amplifications were carried out in a thermocycler Mastercycler (Eppendorf, Hanburg, Germany) using the following program: 40 cycles at 94°C for 1 min, 50°C for 1 min and 72°C for 2 min. An extension step at 72°C for 7 min was added.

The amplified fragments were separated in 1.5% agarose gel, in TAE buffer (0.1 M EDTA pH 8.0, 0.04 M TRIS pH 8.0, and 0.02 M acetic acid), under electrophoresis at 80 V for approximately 3 hours. The amplification products were analyzed under UV light using a Gel Logic 200 Imaging System photo documentation system.

REP primers:

The amplification reactions were conducted as follows: 50 ng of DNA, 3 mM MgCl_2_, 20 mM Tris, 50 mM KCl, 250 μM of dNTP, 1 μM of each primer and two units of Taq DNA polymerase, with a final volume of 20 μL.

The sequences of primers designed by Versalovic et al. (1994) were:

- REP1 5′-IIIICGICGICATCIGGC-3′
- REP2 5′-ICGICTTATCIGGCCTAC-3′

REP amplification used the following program: 5 min at 94°C, followed by 41 cycles at 94°C for 1 min, 45°C for 1 min, and 72°C for 2 min. A final extension step was at 72°C for 7 min was added.

The amplified fragments were separated in 1.2% agarose gel in TAE buffer (0.1 M EDTA pH 8.0, 0.04 M TRIS pH 8.0, and 0.02 M acetic acid) and held the race of 80 V for approximately 3 hours. The amplification of the products were analyzed under UV transilluminator, and filed using a Gel Logic 200 Imaging System photo documentation system.

### Evaluation of molecular data

The amplification products were transformed into binary matrixes where 1 (one) was attributed to the presence of the band and 0 (zero) for absence.

### Analysis of genetic diversity

The genetic relationships between strains were evaluated using a matrix of genetic distances constructed using the complement of the Jaccard similarity coefficient (CSJ), that does not consider negative similarities and the absence of the product (Cruz & Carneiro, 2006).

From estimates of the dissimilarities, the strains were grouped using hierarchical UPGMA method (Unweighted Pair-Group Mean Average) with the test of bootstrap (1000 times) to evaluate the consistency of the group (Efron & Tibshirani, 1993). A second analysis was performed using the pooling method of Tocher. All these tests were performed using the Genes program (Cruz, 2001).

## Results

**Figure 1:**
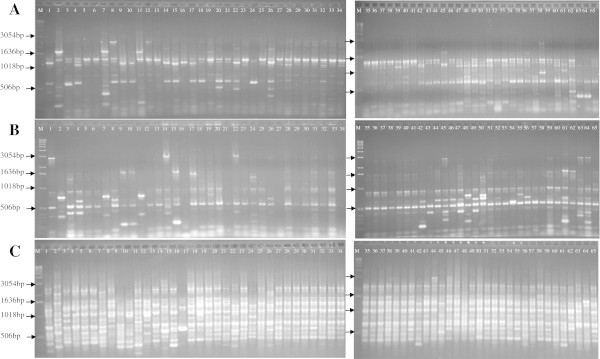
**Rep-PCR fingerprint patterns of** ***B. thuringiensis*** **and 65 references strains. (A)** REP; **(B)** ERIC; **(C)** BOX and **(M)** 50 bp DNA ladder.

The number of fragments using REP primers varied from 1 to 4 per strain, with a total of 10 distinct fragments with the size ranging between 396 to 3.054 bp (Figure 1A). ERIC primers generated between 1 and 9 fragments per strain with a total of 19 distinct fragments ranging from 220 and 2.036 bp (Figure 1B). However, BOX primer generated between 5 and 14 fragments, and a total of 26 distinct fragments between 200 and 3.054 bp (Figure 1C). BOX primers were the most informative among the three pairs of primers used followed by ERIC and REP primers, respectively.

Most of the isolates with subspecies identification showed a distinct profile for the three sequences analyzed (ERIC, REP, and BOX), however isolates 462A (Figure 1, well 5) and 474 (Figure 1, well 6) both subspecies *B. thuringiensis* subsp. *galleriae* showed the same bands for the three sequences analyzed (ERIC, REP and BOX), and the isolates 348 L (well 7, Figure 1) and HD29 (well 8, Figure 1) from the same subspecies showed different profiles. The same results were generated for the subspecies *B. thuringiensis* subsp. *tolworthi* where isolates 344 (well 9, Figure 1) and T09 (well 10, Figure 1) showed the same profile for REP and ERIC, and similar profile when BOX primers were used, however isolates 426 (Figure 1, well 11) and 461A (Figure 1, well 12) showed different profiles. *B. thuringiensis* subsp. *alesti* (well 1 and 2, F. 1), subsp. *aizawai* (well 2 and 4, Figure 1) and subsp. *kurstaki* (well 20 and 21, Figure 1) displayed different profiles for the 3 primer sequences used. Each one of all the other subspecies analyzed amplified a very specific fragment. Among the other 39 *B. thuringiensis* strains with no subspecies identification, those that were found in Goiás State showed similar profiles. However, isolates found in other Brazilian States showed different profiles.

**Figure 2:**
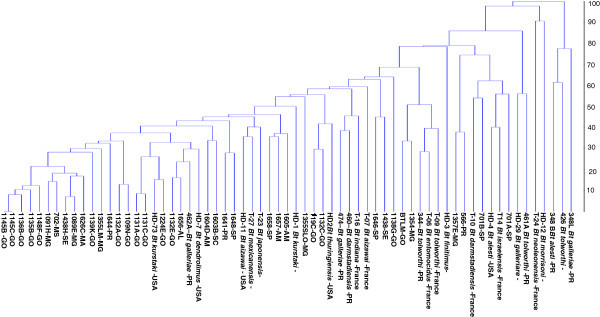
**Dendrogram obtained by using Rep-PCR on purified DNA from** ***B. thuringiensis*** **species followed by evaluation using UPGMA clustering method.**

A total of 55 fragments were generated when ERIC, REP and BOX primers were analyzed together and a dendrogram was generated when UPGMA grouping analysis was used (Figure 2), and considering 45% the average distance (cutting bridge) of the population 10 groups were formed, and group one consisted of 41,5% of the strains, including 2 USDA strains (HD73 *Bt. thuringiensis* subsp*. kurstaki* and HD7 *B. thuringiensis* subsp. *dendrolimus*), and one isolate from Embrapa (462A *B. thuringiensis* subsp*. galleriae*) all the other isolates are from Embrapa with no identification to subspecies. Although 33, 8% of the remaining strains are distributed in the other groups, 24,6% of the strains analyzed were not in any group. Isolates 1145B and 1145C showed the smallest genetic distance (0%), however strains T24 (*Bt neoleonensis*) and HD12 (Bt *morrisoni*) were 90% distant. The groups 3, 5, 6, 7 and 9 are only composed with strains from Embrapa Bt Collection, and group 5 was composed of strains sampled in Paraná State.

A genetic distance of 45% was detected in 53,2% of the strains from Embrapa, and they are in the same group, with 29,8% of the other strains are distributed in different groups, and 17,0% of the isolates appeared to be distinct. With the exception of 344 and T9 strains, both *tolworthi* subspecies, we can observe in the dendogram that 2 *B. thuringiensis* subsp *alesti*, *aizawai* (2), *darmstadiensis* (2), *kurstaki* (2), *galariae* (4) and *tolworth i* (4) showed genetic distance above 45%.

## Discussion

There are not much information published using rep-PCR to study the genetic diversity of *B. thuringiensis* isolates. Some papers describe the comparison of the amplification profiles by each primer pair (Cherif et al. 2003; Sauka et al., 2010), and some researchers discuss the grouping analysis using the individual data of each primer pair (Lima et al. 2002; Mehta et al. 2001). In our research the genetic diversity was studied using group analysis with data obtained when ERIC, REP and BOX primers were used altogether.

This research showed that when ERIC, REP and BOX are used together, the profiles generated are not related to the subspecies of the *B. thuringiensis* isolates, since there were bands in the profile very similar to some isolates of the same subspecie. The similarity of *B. thuringiensis* strains found in Goiás State may be related to the geographical localization (samples were harvested no more than 200 Km apart), and all of them caused 100% in *S. frugiperda* larvae (Valicente and Barreto 2003). However, further studies are needed to confirm the hypothesis that similarity is related with geographic region. Isolates found in the same place may have a higher chance to harbor the same genetic material, however strains found in Goiás State were isolated in four different geographical regions. The remaining strains (collected in other states) represent a small sample of *Bacillus thuringiensis* to make a conclusion. Our results suggest that it is necessary a larger number of isolates but well distributed per region and with different mortality rates against a target insect, aiming to find a similarity between places or mortality. Strains with different mortality rates against *S. frugiperda* (Table 1) were found in the same group. Strains T09 and 344 subsp *tolworthi* were in the same group and the other subspecies did not show similarity for any group. *B. thuringiensis* isolates found in Goiás State showed higher similarity, however isolates found in other States showed a higher genetic distance in relation to the isolates found in Goiás State. Our results suggest that a genetic relationship among *B. thuringiensis* strains it is not defined only by the toxicity of the strain, but for many other reasons that some patterns may be revealed by REP-PCR technique are reproducible, and once these reactions were repeated to check the reproducibility of these fragments, and this reproducibility makes this technique more reliable than RAPD.

## References

[CR1] Cherif A, Brusetti L, Borin S, Rizzi A, Boudabous A, Khyami-Horani H, Daffonchio D (2003). Genetic relationship in the *Bacillus cereus* group by rep-PCR fingerprinting and sequencing of a *Bacillus anthracis*-specific rep-PCR fragment. J Appl Microbiol.

[CR2] Cruz CD (2001). Programa genes: versão windows; aplicativo computacional em genética e estatística.

[CR3] Cruz CD, Carneiro PCS (2006). Modelos biométricos aplicados ao melhoramento genético: 2. ed. rev.

[CR4] Efron B, Tibshirani R (1993). An introduction to the bootstrap.

[CR5] Gilson E, Clément JM, Brutlag D, Hofnung M (1984). A family of dispersed repetitive extragenic palindromic DNA sequences in *E. coli*. EMBO J.

[CR6] Glare TR, O’Callaghan M (2000). Bacillus thuringiensis: biology.

[CR7] Higgins CF, Ames GFL, Barnes WM, Clements JM, Hofnung M (1982). A novel intercistronic regulatory element of prokaryotic operons. Nature.

[CR8] Lima ASG, Guidelli AM, Abreu IL, Lemos MVF (2002). Identification of new isolates of *Bacillus thuringiensis* using rep-PCR products and δ-endotoxin electron microscopy. Genetics and Mol Biolog.

[CR9] Louws FJ, Rademaker JLW, De Bruijn FJ (1999). The three Ds of PCR-based genomic analysis of phytobacteria: diversity, detection and disease diagnosis. Annu Rev Phytopathol.

[CR10] Martin B, Humbert O, Camara M, Guenzi E, Walker J, Mitchell T, Andrew P, Prudhomme M, Alloing G, Hakenbeck R, Morrison DA, Boulnois GJ, Claverys JP (1992). A highly conserved repeated DNA element located in the chromosome of *streptococcus pneumonia*. Nucleic Acids Res.

[CR11] Mehta A, Leite RP, Rosato YB (2001). Assessment of the genetic diversity of *Xylella fastidiosa* isolated from citrus in Brazil by PCR-RFLP of the 16S rDNA and 16S-23S intergenic spacer and rep-PCR fingerprinting. Antonie van Leeuwenhoek.

[CR12] Miralles MP, Peres VJ, Bravo A, Ceron J (2004). Aislamiento y establecimento de una colección de *Bacillus thuringiensis*. Bacillus thuringiensis em el control biológico.

[CR13] Rademaker JLW, De Bruijin FJ, Caetano-Anollés G, Gresshoff PM (1997). Characterization and classification of microbes by rep-PCR genomic fingerprinting and computer assisted patterns analysis. DNA Markes: Protocols, Applications and Overviews.

[CR14] Sauka DH, Basurto-Ríos RE, Ibarra JE, Benintende GB (2010). Characterization of an argentine isolate of *Bacillus thuringiensis* similar to the HD-1 strain. Neotrop Entomol.

[CR15] Shangkuan YH, Chang YH, Yang JF, Lin HC, Shaio MF (2001). Molecular characterization of *Bacillus anthracis* using multiplex PCR, ERIC-PCR and RAPD. Lett Appl Microbiol.

[CR16] Sharples GJ, Lloyd RG (1990). A novel repeated DNA sequence located in the intergenic regions of bacterial chromosomes. Nucleic Acids Res.

[CR17] Shuhaimi M, Ali AM, Saleh NM, Yazid AM (2001). Utilisation of eterobacterial repetitive intergenic consensus (ERIC) sequence-based PCR to fingerprint the genomes of *Bifidobacterium* isolates and other probiotic bacteria. Biotechnol Lett.

[CR18] Stern MJ, Ames GF, Smith NH, Robinson EC, Higgins CF (1984). Repetitive extragenic palindromic sequences: a major component of the bacterial genome. Cell.

[CR19] Valicente FH, Barreto MR (2003). *Bacillus thuringiensis* survey in Brazil: geographical distribution and insecticidal activity against *Spodoptera frugiperda* (J.E. Smith) (Lepidoptera: Noctuidae). Neotrop Entomol.

[CR20] Valicente FH, Fonseca MM (2004). Susceptibilidade da lagarta-do-cartucho do milho, *Spodoptera frugiperda*, a diferentes isolados de *Bacillus thuringiensis*. Revista Brasileira de Milho e Sorgo.

[CR21] Versalovic J, Koeuth T, Lupski JR (1991). Distribution of repetitive DNA sequences in eubacteria and application to fingerprinting of bacterial genomes. Nucleic Acids Res.

[CR22] Versalovic J, Schneider M, De Bruijn FJ, Lupski JR (1994). Genomic fingerprinting of bacteria using repetitive sequence-based polymerase chain reaction. Methods in Molecular and Cellular Biology.

[CR23] Vilas-Boas G, Sanchis V, Lereclus D, Lemos MVF, Bourguet D (2002). Genetic differentiation between sympatric populations of *Bacillus cereus* and *Bacillus thuringiensis*. Appl Environ Microbiol.

[CR24] Yamamoto T, Dean DH, Charles JF, Delécluse A, Nielsen-Le Roux C (2000). Insecticidal proteins produced by bacteria pathogenic to agriculturas pests. Entomopathogenic bacteria: from laboratory to field application.

